# Anti-doping Policy, Therapeutic Use Exemption and Medication Use in Athletes with Asthma: A Narrative Review and Critical Appraisal of Current Regulations

**DOI:** 10.1007/s40279-019-01075-z

**Published:** 2019-03-18

**Authors:** Hayden Allen, Susan H. Backhouse, James H. Hull, Oliver J. Price

**Affiliations:** 1 Carnegie School of Sport, Leeds Beckett University, Leeds, LS6 3QT UK; 2 Department of Respiratory Medicine, Royal Brompton Hospital, London, UK; 3 National Heart and Lung Institute, Imperial College London, London, UK

## Abstract

Asthma is prevalent in athletes and when untreated can impact both respiratory health and sports performance. Pharmacological inhaler therapy currently forms the mainstay of treatment; however, for elite athletes competing under the constraints of the World Anti-Doping Code (Code), a number of established therapies are prohibited both in and/or out of competition and/or have a maximum permitted dose. The recent release of medical information detailing inhaler therapy in high-profile athletes has brought the legitimacy and utilisation of asthma medication in this setting into sharp focus. This narrative review critically appraises recent changes to anti-doping policy and the Code in the context of asthma management, evaluates the impact of asthma medication use on sports performance and employs a theory of behaviour to examine perceived determinants and barriers to athletes adhering to the anti-doping rules of sport when applied to asthma.

## Key Points

**Table:** 

The perception that asthma medication may enhance sports performance has created a negative stigma towards athletes with asthma, inhaler therapy and therapeutic use exemptions (TUEs).
The capability, opportunity, motivation—behaviour (COM-B) model is a theoretical starting point to understanding behaviour in this setting and provides foundations for intervention development (e.g. education programmes and environmental restructuring).
Future developments in policy and practice have the potential to change behaviour, establish trust in the anti-doping system, and in turn, alter the attitudes and perceptions of asthma medication use in sport.

## Introduction

Asthma is frequently reported as the most common medical condition in elite-level athletes [1–3], with recent studies indicating a prevalence of 25–75% in susceptible cohorts [4–6]. The reason for the heightened incidence in elite sport remains to be fully established; however, there is now evidence indicating that airways hyper-reactivity can develop over the course of a sporting career (for review see Price et al. [7]). Beyond the elite athlete population, an increased frequency of asthma has also recently been reported in UK-based recreational athletes (~ 15%) when compared with the general population [8].

For the most part, the treatment of asthma and/or exercise-induced bronchoconstriction (EIB) in athletes is well established, with the recommendation that a short-acting β-2 agonist (SABA) (e.g. salbutamol) forms the mainstay of pharmacological therapy [9, 10]. However, for athletes competing under the constraints of the World Anti-Doping Code (Code), the use of SABA and several other commonly prescribed asthma medications puts athletes at risk of returning an adverse analytical finding (AAF), potentially leading to an anti-doping rule violation (ADRV) and a period of ineligibility from sport [11]. Prior to initiating treatment, it is therefore imperative that athletes and their support personnel (e.g. sports physicians) have a thorough understanding of the Code and the annually updated World Anti-Doping Agency (WADA) prohibited list [12].

In keeping with their non-athletic counterparts, elite athletes with asthma are also susceptible to acute illness (e.g. respiratory tract infection) or ‘exacerbations’ that often require additional pharmacological therapy, which may be prohibited in competition, to restore and optimise health [13]. To ensure health protection is afforded for athletes bound by anti-doping rules and regulations, the International Olympic Committee—Medical Commission (IOC-MC) introduced a policy in the 1980s for permitted use of prohibited substances and methods [14]. Currently, athletes competing at the elite level are thus typically required to provide objective evidence of asthma before a therapeutic use exemption (TUE) may be granted to permit use of an otherwise prohibited substance or medication dose.

The process to obtain a TUE was originally formalised following the introduction of the International Standard for Therapeutic Use Exemptions [15]. However, the legitimacy and utilisation of asthma medication use in this setting has been questioned for some time and highlighted in recent years following the release of personal medical information of several high-profile athletes by Russian cyber-espionage group Fancy Bears. For many this has served to reinforce perceptions of wrong-doing within the athlete community [16] and may prompt the misuse of asthma medication amongst those potentially seeking to gain an advantage.

Whilst instinctively, a decision to comply with the Code may be viewed as straightforward, factors underpinning non-compliance are often complex and have recently been conceptualised in the scope of a model evaluating the multi-faceted *dopogenic* environment [17]. In this model, it is proposed that an athlete may be influenced by the surroundings, opportunities and conditions that promote ADRVs. To fully understand the drivers of the misuse of asthma medication in sport, an appraisal of current literature guided by a contemporary and overarching model of behavioural theory (the capability, opportunity, motivation—behaviour [COM-B] model) [18] is required. Drawing upon this model, it is proposed that behaviour (B) is the result of an interaction between three necessary conditions: capability, opportunity and motivation. For an individual to engage in a specific behaviour (B) they must have the psychological and physical capability (C) (e.g. knowledge), the social (e.g. peers) and physical opportunity (O) (e.g. resources), and the motivation (M) to undertake the behaviour. Motivation covers automatic processes, such as habit and impulses, as well as reflective processes, such as intention and choice [18].

This narrative review critically appraises recent changes to anti-doping policy and the Code in the context of asthma management, evaluates the impact of asthma medication use on sports performance, and employs a theory of behaviour (COM-B) to examine perceived determinants and barriers to athletes adhering to the anti-doping rules of sport when applied to asthma. In order to achieve these objectives, publications in the peer-reviewed literature from January 2004 (conception of the Code) until December 2018 were reviewed using search terms such as ‘asthma’, ‘exercise-induced asthma or bronchoconstriction’ in combination with ‘athletes’, ‘anti-doping’, ‘medication’ and ‘sports performance’.

## The World Anti-Doping Code

WADA was established in 1999 to harmonise global anti-doping policy and practice. Most countries, and almost all sports, are signatories to the Code, with the major exceptions being North American professional sporting bodies (e.g. Major League Baseball). First published in January 2004 [19], the Code provides the framework for anti-doping polices, rules and regulations within sport organisations and among public authorities. Along with five international standards (e.g. International Standard for Therapeutic Use Exemptions; List of Prohibited Substances and Methods), the Code serves to ensure that anti-doping policies and procedures are the same for all athletes and support personnel. Updated annually, the prohibited list contains substances and methods that if detected in the absence of a TUE, will result in an ADRV. The International Standard for Therapeutic Use Exemptions states that an athlete will only be granted a TUE if the following conditions are met: (a) the athlete would experience significant health impairment if the prohibited substance or method were to be withheld, (b) therapeutic use of the prohibited substance or method is unlikely to produce any additional performance enhancement, (c) there is no reasonable therapeutic alternative to the use of the prohibited substance or method, and (d) the necessity for the use of the prohibited substance or method is not a consequence of the prior use (without a TUE) of a substance or method that was prohibited at the time of use [15].

### The Code and Asthma

Following the conception of the Code in 2004, all inhaled β-2 agonists were prohibited without an abbreviated TUE (i.e. written medical notification) submitted to authorise the use of inhaled SABA (i.e. salbutamol and terbutaline), inhaled long-acting β-2 agonists (LABA) (i.e. formoterol and salmeterol) and inhaled corticosteroids [19]. Due to concerns over unnecessary β-2 agonist use in elite sport [20], the prohibited list was updated in 2009 resulting in all forms of β-2 agonists prohibited without an authorised TUE [21]. Since this point, athletes have been required to provide comprehensive medical history with supporting objective evidence of asthma via bronchodilator reversibility or bronchoprovocation challenge testing to obtain a TUE [22] (summarised for reference in Table 1).

**Table 1:** Objective testing accepted by the World Anti-Doping Agency to diagnose asthma in athletes

Diagnostic methods	Criteria
Bronchodilator reversibility	≥ 12% increase in FEV_1_^a^
Bronchoprovocation challenge(s):	
Direct	
Methacholine/histamine	≥ 20% reduction in FEV_1_
Indirect	
Exercise challenge (laboratory and field-based)	≥ 10% reduction in FEV_1_^c^
Eucapnic voluntary hyperpnoea (EVH)^b^	≥ 10% reduction in FEV_1_^c^
Dry powder mannitol	≥ 15% reduction in FEV_1_
Hypertonic saline (4.5%)	≥ 15% reduction in FEV_1_

*FEV*_*1*_ forced expiratory volume in one second, *IOC*-*MC* International Olympic Committee—Medical Commission

^a^Supports asthma diagnosis

^b^Optimal test to detect asthma in athletes (IOC-MC)

^c^Sustained reduction in FEV_1_ required (i.e. two consecutive timepoints) to confirm diagnosis

Permitted limits were introduced for inhaled salbutamol, salmeterol and formoterol between 2010 and 2012. The requisite to submit a TUE during this period was therefore no longer required for these substances (unless an athlete exceeded permitted limits in a medical emergency whereby a retroactive TUE was still required) [23]. The decision to implement threshold values was made to remove the administrative burden of TUE approval, and coincided with limited evidence concerning performance-enhancing properties associated with inhaled β-2 agonist therapy [24]. Following these modifications, in 2013, the maximum permitted dose for inhaled formoterol was updated to 54 µg over 24 h [25] and, 4 years later, the maximum permitted dose for inhaled salbutamol and inhaled salmeterol, over a 24-h period, was updated to 1600 µg (not exceeding 800 µg per 12 h) and 200 µg, respectively [26].

The current prohibited list (summarised for reference in Table 2) states that athletes are permitted to administer inhaled salbutamol (in divided doses not exceeding 1600 µg in 24 h and 800 µg in 12 h), formoterol (≤ 54 µg in 24 h) and salmeterol (≤ 200 µg in 24 h) [12] without a TUE. Salbutamol and formoterol are associated with permitted urine thresholds of 1000 ng/mL and 40 ng/mL, and decision limits (accounting for measurement uncertainty) of 1200 ng/mL and 50 ng/mL, respectively [27]. An athlete found to exceed the urinary decision limit for a substance may request an individualised pharmacokinetic study. Indeed, following recent high-profile anti-doping investigations concerning asthma medication use in elite athletes, a pharmacokinetic modelling study demonstrated that an AAF for salbutamol has the potential to occur irrespective of adherence to current guidelines (i.e. doses administered below or within upper limits) [28]. To date, a urine threshold for salmeterol  has not been implemented despite evidence concerning the ability to detect following inhalation [29]. Similarly, although differences in urinary concentrations between oral and inhaled terbutaline have been demonstrated, a threshold has yet to be established [30]. The current clinical practice guideline statement concerning the management of EIB in athletes recommends inhaled SABA—15 min prior to commencing exercise—as the most effective approach to managing troublesome respiratory symptoms [9]. However, in the context of elite sport, athletes typically complete multiple exercise bouts or training sessions per day. To avoid overuse and potential adverse effects, or reduced tolerance and efficacy of reliever medication [31], it is recommended that daily inhaled corticosteroid maintenance therapy is initiated to target underlying airway inflammation and optimise asthma management [9]. Inhaled corticosteroids remain permitted in and out of competition without a TUE [12]. However, the systemic administration of corticosteroids (i.e. oral route most commonly), that may be used to treat severe acute asthma exacerbations [13] requires a TUE for use in competition but is not prohibited out of competition [12].

**Table 2:** Asthma medications and the prohibited list (2019)—status and impact on sports performance

Asthma medication	Prohibited list status	Impact on sports performance
β-2 agonists		
*Short*-*acting β*-*2 agonists* Salbutamol (excluding inhaled) Reproterol Terbutaline	Prohibited (all selective and non-selective β-2 agonists, including all optical isomers)	Increased strength and sprint power following acute and chronic administration [44–47, 51–53] Improvement in submaximal endurance performance [50]
*Long*-*acting β*-*2 agonists* Salmeterol (excluding inhaled) Formoterol (excluding inhaled) Indacaterol Olodaterol Tulobuterol Vilanterol	Prohibited (all selective and non-selective β-2 agonists, including all optical isomers)	No data available
*Other (intermediate acting)* Fenoterol Higenamine Procaterol	Prohibited (all selective and non-selective β-2 agonists, including all optical isomers)	No data available
Inhaled salbutamol	Permitted (maximum 1600 µg over 24 h in divided doses not to exceed 800 µg over 12 h)	No evidence to support improvement in aerobic capacity [34] or endurance performance [35–39]
Inhaled formoterol	Permitted (maximum delivered dose of 54 µg over 24 h)	Improved sprint performance [42]
Inhaled salmeterol	Permitted (maximum 200 µg over 24 h)	
Corticosteroids Betamethasone Budesonide Cortisone Deflazacort	Prohibited in-competition only (systemic administration [i.e. oral, intravenous, intramuscular and rectal] of corticosteroids)	Improved time to exhaustion at sub-maximal intensities (~ 70% aerobic capacity) following acute [54, 55] and short-term administration [56–58]
Dexamethasone Fluticasone Hydrocortisone Methylprednisolone Prednisolone Prednisone Triamcinolone	Permitted at all times (inhaled administration of corticosteroids)	No impact on endurance performance [43]

It is important to acknowledge that any responsible clinician should ensure that the care afforded to an athlete with asthma is always prioritised. In the event a prohibited substance is administered to treat an asthma exacerbation, the athlete is required to apply for a retroactive TUE [15]. A retroactive TUE may also be sought by drug-tested athletes who are not deemed to be International Level or National Level if a doping control test returns an AAF [11]. In this scenario, guidance provided by WADA to clinicians emphasises the need for “full and clear documentation of the medical incident” [22].

## Impact of Medication on Sports Performance

The impact of asthma and associated treatment on athletic performance has been extensively investigated (for review see Price et al. [32]). Yet, despite several proposed physiological mechanisms indicating asthma may impair sporting performance, there remains limited experimental evidence to support or refute this concept. Indeed, elite-level athletes with asthma are consistently reported to match and indeed in some cases out-perform their non-asthmatic rivals [1], fuelling widespread speculation concerning the performance-enhancing properties of asthma therapy [33].

### Inhaled β-2 Agonists and Corticosteroids Not Requiring a Therapeutic Use Exemption (TUE)

A great number of studies have been undertaken evaluating the impact of inhaled salbutamol on exercise performance with no clear benefit demonstrated [34–37] (Table 2). Similarly, aerobic exercise performance appears to remain unchanged following the administration of inhaled LABA [38, 39]. Over the past decade, multiple systematic reviews have concluded that inhaled β-2 agonists yield limited ergogenic benefit [24, 40, 41]; however, it is important to note that inhaled combination therapy (i.e. salbutamol, formoterol and salmeterol) (each within permitted doses) has been reported to improve sprint performance and maximal voluntary contraction [42]. To date, studies investigating inhaled corticosteroids at therapeutic doses have failed to show any improvement in exercise performance [43].

### Inhaled/Oral β-2 Agonists and Corticosteroids Requiring a TUE

Several studies investigating high-dose terbutaline have shown improvements (2–8%) in peak and average sprint power in trained cyclists [44, 45], as well as meaningful improvements in time to exhaustion at near maximal power outputs when combined with an inhaled corticosteroid [46]. Furthermore, daily terbutaline administration has been shown to elicit a significant increase in skeletal muscle growth in healthy males irrespective of a concurrent resistance exercise programme [47]. On the contrary, chronic use of terbutaline has been reported to impair skeletal muscle adaption following high-intensity training [48]. Despite this, establishing a urine threshold for athletes who acquire a TUE for terbutaline has been proposed to reduce supra-therapeutic dosing and the potential for performance enhancement [49]. The short-term oral administration of salbutamol has been shown to significantly improve sub-maximal (~ 70–80% maximal oxygen uptake [*V*O_2max_]) time to exhaustion [50], with improvements in strength and power also noted [51, 52]. Furthermore, oral salbutamol has recently been shown to increase protein turnover rates in skeletal muscle following resistance exercise [53].

The acute administration of an oral corticosteroid has been previously shown to improve prolonged sub-maximal exercise performance in trained cyclists [54, 55]. Improvements in time to exhaustion at sub-maximal exercise intensities (70–75% *V*O_2max_) have also been observed following systemic corticosteroid administration [56–58]. It has been proposed that oral corticosteroids may improve exercise performance from both a psychological and physiological perspective by inducing the perception of euphoria [59] and increasing fat oxidation to meet energy requirements during exercise [60]. Oral corticosteroids have also been associated with increased lipolysis [61], resulting in changes to body composition; the latter being considered desirable for endurance-based athletes (i.e. increased power: weight ratio) [62]. Finally, a blunted pro-inflammatory response post-exercise has also been observed following oral corticosteroid administration [55], which in turn may translate to enhanced recovery between repeated exercise bouts (e.g. tennis tournaments or multiple-stage cycling events etc.).

## Asthma Medication Use in Athletes—Treatment or Permitted Doping?

In recent major sporting competitions such as the Olympic Games, World and European Championships and Commonwealth Games, asthma has been noted as a common justification for the use of prohibited substances by elite athletes, illustrated by the leaking of TUE information and medication use by the Russian cyber espionage group ‘Fancy Bears’ [63]. Although there was no suggestion of wrong-doing on the part of the athletes whose data was leaked, the omnipresent use of asthma medication by high-profile athletes questions the legitimacy of the anti-doping system in this setting [33].

Fundamentally, the purpose of the Code is not to restrict the use of required medication in athletes with asthma and prevent them from becoming elite competitors, however abuse of this system is both undesirable and certainly unethical. Media headlines covering high-profile athletes’ use of asthma medication may encourage the misuse of inhaler therapy amongst sub-elite or recreational-level athletes seeking a competitive advantage. On the contrary, the negative stigma surrounding asthma medication may actually act to deter an athlete from disclosing their diagnosis and/or refrain from using prescribed medication due to fear of being labelled a cheat. Taken together, these behaviours may be detrimental to the overall health and well-being of athletes or individuals partaking in sport across all levels. Understanding athlete (and associated support personnel) capability, opportunity and motivation with regards the current TUE system and use of asthma medication is a necessary first step to facilitate interventions and modifications to ensure global anti-doping policy and practice can be reviewed and effectively delivered in a supportive and progressive manner.

### Capability (Knowledge and Understanding)

Under the Code and concept of ‘strict liability’, athletes are solely responsible for the substances detected in their biological system regardless of whether use is intentional or not [11]. Therefore, athletes need to be knowledgeable and comply with all applicable anti-doping rules and regulations [11]. However, recent studies have exposed partial knowledge and understanding of the policies and rules that govern participation in sport [64], rendering athletes at increased risk of committing ADRVs. For example, in a study involving athletes from the UK, USA, Australia and Canada, the prohibited list status of substances found in over-the-counter medications was correctly identified in only 35% of cases presented [65]. This finding is notable as 66% of the survey respondents had been subject to in- or out-of-competition testing. This lack of capability to navigate the complex anti-doping landscape can be linked to athletes’ insufficient exposure to formal anti-doping education [64]. Compounding this situation further, stigma attached to anti-doping information seeking within elite sporting organisations has previously been reported [66].

Athletes reporting breathing difficulty most often seek medical guidance from non-specialist (i.e. neither respiratory nor sports medicine) healthcare professionals to manage their medical condition [67, 68]. It is important that all clinicians are aware and remain up to date with asthma guideline reports [9] given the global prevalence of the condition, but specifically to ensure diagnosis is robust [69]. Over the past two decades, a wealth of published research has supported the concept that asthma is frequently misdiagnosed (i.e. over- and under-detected) in both elite and recreational athletes [8, 70]. Despite recognition of the disconnect between self-report respiratory symptoms and objective evidence of asthma [71], a study by Hull et al. found that approximately one-quarter of primary care clinicians in the UK initiate treatment based on clinical history alone [68]. Although objective testing is often requested, test selection is typically sub-optimal for the assessment of the breathless athlete (e.g. baseline spirometry and/or peak expiratory flow) [68]. To date, the most appropriate diagnostic test and/or criteria employed to detect asthma in athletes remains debated [72–74]; however, it has been recognised for some time that (a form of) bronchoprovocation challenge is required to support a diagnosis in the absence of baseline airflow obstruction with reversibility (Table 1). In support of this concept, and pertinent to anti-doping, a retrospective analysis in elite Portuguese athletes reported that the number of TUE applications for asthma medication decreased by over half (51%) between 2008 and 2009 following a mandated requirement to objectively document asthma [75]. Despite these findings, the diagnostic test currently endorsed by the IOC-MC (i.e. indirect bronchoprovocation via eucapnic voluntary hyperpnoea) [76] remains under-utilised and largely overlooked [68].

For clinicians prescribing asthma medication, an appreciation and understanding of evidence-based treatment strategies to optimise management remains a priority. However, in the same study by Hull et al. it was also reported that two-thirds of clinicians were unsure of the medications a competitive athlete is legally permitted to use following a diagnosis [68]. The annual updating of the prohibited list only adds to the challenges faced by clinicians when prescribing medications to athletes competing under the Code. The lack of referring for specialist testing and knowledge of the Code is likely attributed to (a) the challenges of disseminating research to the relevant wider audience (e.g. sports physicians), (b) translation of findings into clinical practice, (c) limited access to appropriate diagnostic methods and (d) cost of referral to centres offering specialist assessment.

Taken together, the capability of athletes and clinicians (defined as athlete support personnel under the Code) to comply with current rules and regulations appears compromised, increasing the potential to commit an ADRV [67]. To support athletes and the medical profession, tailored and targeted education programmes for clinicians therefore need to be developed and delivered to help rectify this situation.

### Opportunity (Environment and Resources)

To address the concern surrounding clinician capability in the future, increasing the number of referrals to sports medicine practitioners and/or respiratory specialists (e.g. technicians and physiologists) with expertise in the diagnosis and management of breathing disorders in athletes may ensure optimal diagnostic methods and test interpretation. To ensure this happens, referral for specialist services must be easily available and promoted widely across the sports medicine community. If specialist services are not accessed, unnecessary inhaler therapy or the illegitimate prescription of asthma medication may be afforded to athletes. This is concerning given that the adverse health implications of unnecessary chronic SABA administration have been recognised for some time [31].

In some environments, the physical access to healthcare professionals willing to undermine the system by authorising a TUE for a fictional illness (e.g. asthma or musculoskeletal injury) has been reported [77]. For example, Lentillon-Kaestner and Carstairs previously documented premeditated misuse of therapeutic substances in a study involving young elite cyclists, with one rider admitting “If we want to take banned substances legally, we can. You just need to know a doctor who provides the therapeutic use exemption rather easily” [77]. Furthermore, in a similar study of Swiss National cyclists, another stated “All the riders I know, they all have tried cortisone. […]. Yes, they take therapeutic use exemptions (TUEs).[…].They play with the rules. It depends what you mean by doping but everyone I know, they do that” [78].

### Opportunity (Social Influences)

Influenced by the *dopogenic* environment, the way athletes, their support personnel and the public interpret TUE use is shaped by the prevailing social norms, including a distrust in competitors’ abuse and/or authorities’ management of the system [16, 64]. For example, amongst a sample of elite Danish athletes, the perception of over half surveyed was that fellow athletes had been granted a TUE without the clinical requirement, with many using higher doses of prescribed medication than required [16]. Similarly, in a cohort of 260 elite athletes from four different sports federations (IAAF: athletics, FIBA: basketball, FIS: skiing, FIVB: volleyball), approximately half considered it unfair that athletes were granted permission to use an otherwise prohibited substance; with one athlete reporting “asthma and other disabilities should never give right to those athletes using a TUE when competing in the same champs” [79]. Furthermore, a recent study by Bourdon and colleagues highlighted that nearly half of their elite athlete cohort (approximately 60% endurance athletes) suspected their peers may abuse the system and that fellow competitors had incorrectly received a TUE [80]. In other sports where doping has received significant attention (e.g. bodybuilding), research has shown that an athlete is more likely to misuse a substance if they believe, or directly observe, others abusing [81]. This likelihood can be explained by moral disengagement, a process whereby an athlete justifies unethical behaviours because of perceived extenuating circumstances. For example, they may justify unethical behaviour on the basis that their peers are abusing the TUE system and getting away with it and/or consider it to be levelling the playing field. Processes such as moral disengagement offer a coping strategy for reducing cognitive dissonance that occurs from holding conflicting beliefs and values. Central to the effects of moral disengagement concerning the TUE system is the training and competition environment [81], most pertinent when applied to groups with high asthma prevalence (e.g. pool-based or winter sport athletes) [7].

This research brings to the forefront the *social opportunity* in which asthma medications and the TUE system are perceived to be a legitimised form of doping. In turn, this may provide athletes and support staff with motivation to engage in behaviours that go against the spirit of sport [11], and that have the potential to compromise athlete health and well-being. Influential others might also provide the motivation for non-asthmatic athletes to use unnecessary asthma medication through the widespread perception that inhaled SABA improves sports performance. As detailed, this perception is despite the fact that the (potentially detrimental) impact of asthma on exercise performance has yet to be fully substantiated [32], with the majority of research supporting the absence of ergogenic benefit for inhaled SABA (i.e. salbutamol) and LABA (i.e. formoterol and salmeterol) that do not currently require a TUE in asthmatic and non-asthmatic athletes.

### Motivation (Beliefs About Consequences)

At the other end of the spectrum, it has been reported that athletes may avoid applying for a TUE despite therapeutic need [16, 80]. Whilst these athletes may have the capability to adhere to a prescribed treatment, the stigma of TUEs and asthma medication may negatively influence an athlete’s motivation to manage their condition therapeutically. This is concerning as the continuation of training and competition without appropriate treatment may lead to a deterioration in condition and possibly sporting performance. In contrast, in recreational athletes, poor asthma control due to the non-adherence to medication may deter physical activity and exercise engagement but may also have more serious consequences, particularly in athletes with severe or uncontrolled asthma (i.e. exacerbation and heightened risk of mortality). Increasing the transparency of medication use and the TUE process may be an important intervention to address some of the issues raised. The rights of athletes concerning their personal data and medical information must also be balanced against the effects that public disclosure of all medication use and granted TUEs may have on perceptions of cheating within the athlete community [82].

## Asthma in Athletes—A Call to Action

**Fig. 1:**
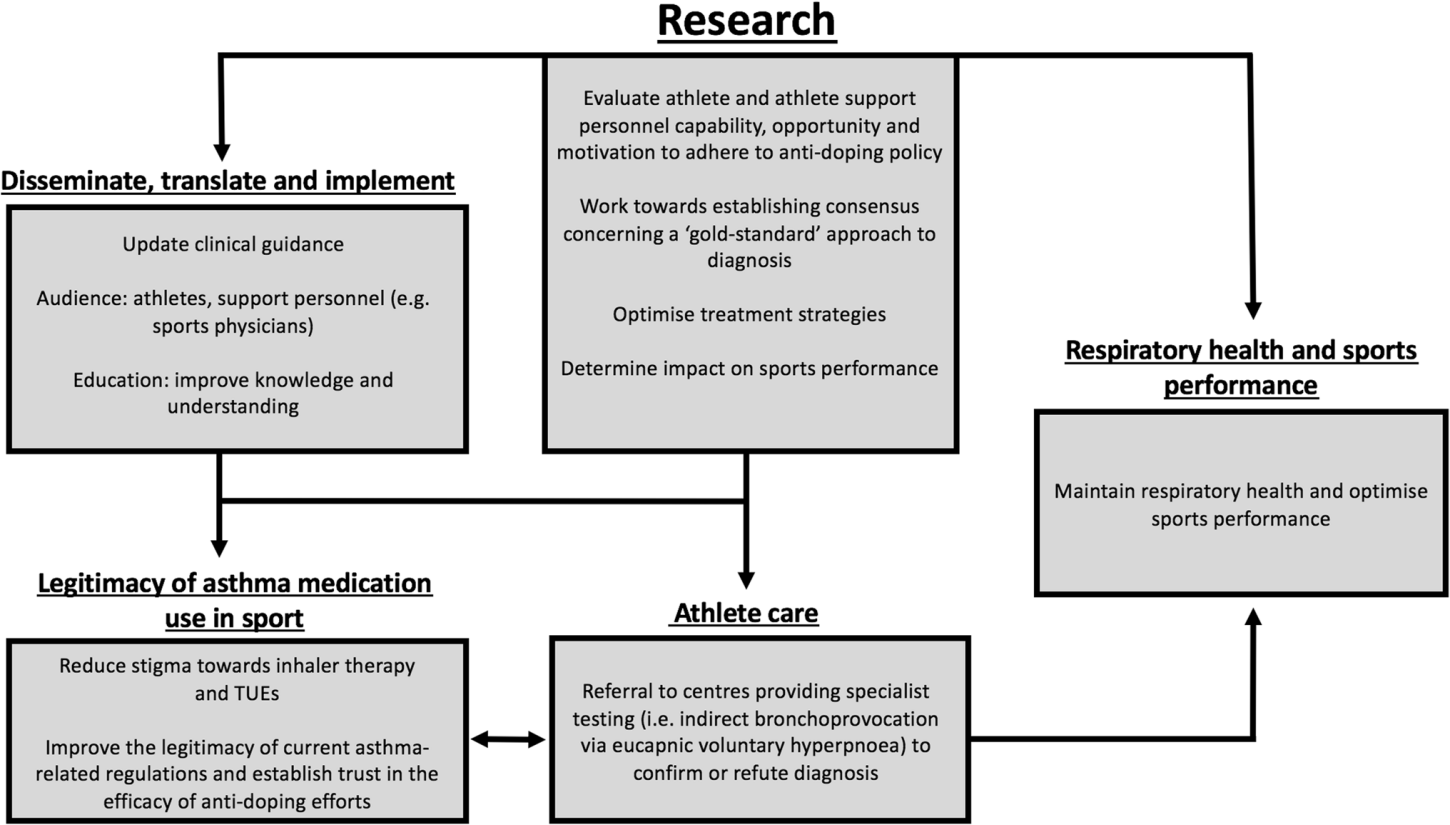
Asthma in athletes—a call to action for future research priorities. *TUEs* therapeutic use exemptions

This narrative review provides the first theory-informed critical appraisal of anti-doping policy as it applies to asthma management, medication use and sports performance. Applying the COM-B model is a theoretical starting point to understand behaviour concerning asthma medication use in sport and we offer this important overview to drive a ‘call to action’ concerning future research priorities (Fig. 1). To date, there remains limited research focusing on athlete knowledge and perceptions of doping and TUEs specific to asthma. It is probable that both athletes and clinicians have inadequate knowledge of current anti-doping policy regarding asthma medication, which heightens the risk of receiving AAF and/or committing an ADRV.

The reliance on clinicians to provide guidance to athletes presenting with respiratory symptoms is also concerning as there appears to be a disconnect between research-informed evidence and current practice. The challenges clinicians face securing a robust diagnosis only furthers the negative stigma towards the use of inhaler therapy in athletes. Although several objective methods of assessment (each with a unique diagnostic methodology) are currently accepted, establishing consensus regarding the ‘gold-standard’ approach to diagnosis (with clear objective criteria), and overcoming challenges accessing centres specialising in athlete respiratory health is necessary to optimise the care afforded to athletes with and without asthma.

To fully understand and recognise the complexity of the *dopogenic* environment in this setting, it is necessary to qualitatively examine athlete motivation to use asthma medication and perceptions and understanding of asthma TUEs in sport. Moreover, the true impact of asthma and associated medication on sports performance remains to be fully determined. Until this point, it is likely that the negative stigma associated with inhaler therapy will remain. Although untested in the context of TUEs, the use of the COM-B model provides foundations for intervention development (e.g. education programmes and environmental restructuring) that can target the salient barriers of Code compliance and reduce the potential for ADRVs. Moving forward, developments in policy and practice have potential to change the behaviour of athletes and athlete support personnel, establish trust in the anti-doping system, and in turn, alter the attitudes towards and perceptions of asthma medication use in sport.
